# Boosting the electrochemical properties of carbon materials as bipolar electrodes by introducing oxygen functional groups†

**DOI:** 10.1039/d0ra06888h

**Published:** 2020-09-24

**Authors:** Yaxiong Zhang, Ying Liu, Yunfei Bai, Yupeng Liu, Erqing Xie

**Affiliations:** Key Laboratory for Magnetism and Magnetic Materials of the Ministry of Education, School of Physical Science and Technology, Lanzhou University Lanzhou 730000 China xieeq@lzu.edu.cn; School of Physics and Optoelectronic Engineering, Ludong University Yantai 264025 China; State Key Laboratory of Applied Organic Chemistry, Key Laboratory of Nonferrous Metal Chemistry and Resources Utilization of Gansu Province, College of Chemistry and Chemical Engineering, Lanzhou University Lanzhou 730000 China

## Abstract

Carbon materials are often used as both positive and negative electrodes (bipolar electrode materials) in energy storage devices, which significantly reduces the preparation complexity of the electrode. Herein, oxygen-modified carbon nanotubes mounted on carbon cloth (CCC) present a high areal capacitance as both positive and negative electrodes in a safe neutral electrolyte. The introduction of oxygen functional groups facilitates the formation of many electrochemical active sites and defects conducive to ion diffusion. When carbon materials are utilized as negative electrodes, the charge storage characteristics are mainly dependent on the adsorption and desorption of the ions (corresponding to the electric double layer capacitance). Whereas, when utilized as positive electrodes, the charge storage characteristics come from the intercalation and de-intercalation of the electrolyte ions in the multi-defect carbon material. The maximum areal capacitance measured at the positive electrode and negative electrode was 336 mF cm^−2^ and 158 mF cm^−2^, respectively. The measured areal capacitance of the assembled symmetrical supercapacitors was 93.6 mF cm^−2^, and the areal energy density reached 33 μW h cm^−2^ at a power density of 793 μW cm^−2^. It is believed that the efficient preparation method and electrochemical mechanism elucidated in this work can guide the practical applications of carbon cloth in supercapacitors.

## Abstract 2

Carbon materials with effective oxygen functional groups as positive and negative electrodes and their special energy storage mechanism.

## Graphical abstract

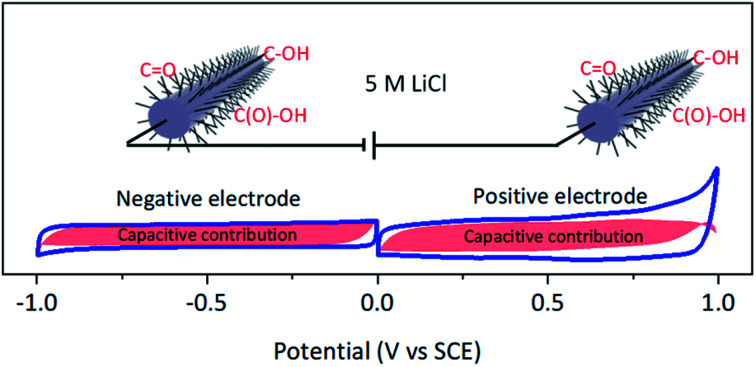

## 1. Introduction

Supercapacitors are promising energy storage devices because of their high power density, environmental friendliness, excellent stability, and low cost. There are many excellent supercapacitor electrode materials, such as carbon materials (activated carbon, carbon nanotubes, carbon cloth, graphene, and carbide-derived carbons, *etc*.), transition metal-based materials (RuO_2_, MnO_2_, NiO, Co_3_O_4_, Fe_2_O_3_, Ni(OH)_2_, Co(OH)_2_, Zn_2_Nb_34_O_87_, Al_0.5_Nb_24.5_O_62_, and HfNb_24_O_62_, *etc*.), and conducting polymers (polypyrrole, polyaniline, and polythiophene, *etc*.).^1–5^ Among them, carbon materials have been shown to exhibit excellent properties as positive electrode materials in acid and as negative electrodes in alkaline media.^6–9^ It is generally accepted that in an acid electrolyte, electric double layer capacitance (EDLC) results primarily from the adsorption of hydrogen ions on the surface of the electrode and partially as a result of redox reaction with nitrogen and other impurity species.^10–13^ In alkaline electrolytes, EDLC results from the adsorption/desorption of electrolyte ions at the surface of the electrode accompanied by additional pseudo-capacitance due to the presence of functional groups.^14–16^ Importantly, in neutral electrolytes, carbon materials can be used as positive and negative electrodes simultaneously, and many symmetric supercapacitor technologies are based on this idea.^17,18^ Despite this, the energy storage mechanism of the positive and negative electrode is not well understood, which limits further improvements to the performance of carbon materials. To thoroughly understand the electrochemical processes of electrode capacitance, Bruce Dunn divided the capacitance into surface-controlled and diffusion-controlled contributions.^19–22^ The magnitude of diffusion-controlled capacitance decreases with increasing scan rate, while surface-controlled capacitance remains constant with increasing scan rate.^23–25^ As a potential energy storage material, it is necessary to understand the charge storage mechanism of carbon-based supercapacitors in order to guide the application of electrodes.

As one of the most common material choices for bipolar electrodes,^26–28^ carbon materials such as graphene, carbon nanotubes (CNTs), activated carbon, and carbon cloth present high electrical conductivities, large specific surface areas, and stable surface chemistry.^29–32^ CNTs specifically are a promising candidate for supercapacitor electrodes, given their high flexibility, potential for large-scale production, and excellent electrical conductivity.^33–35^ However, CNTs grown through chemical vapor deposition (CVD) are hydrophobic, and further activation is required to realise high areal capacitance and hydrophilicity for use in aqueous electrolytes, leading to the complication of the fabrication process. Thus, a simple and effective method of surface modification of CNTs is urgently required. Plasma treatment is an effective and convenient environmentally friendly method to introduce nitrogen, oxygen, and other groups to the surface of CNTs, which can enhance the hydrophilicity and capacitance characteristics.^36–39^ Fei-Hong Kuok and co-workers used a dc-pulse nitrogen atmospheric-pressure plasma jet to process CNTs, achieving an areal capacitance of 5.89 mF cm^−2^ in 2 M KCl.^40^ Hung-Hua Chien and co-workers treated a carbon cloth with a dc-pulse nitrogen atmospheric pressure plasma jet and achieved a capacitance of 106.89 mF cm^−2^.^37^ The studies discussed the excellent electrochemical performance of carbon materials, showing that their application of plasma treatment is a successful method for the surface modification of carbon materials.

In this work, an air plasma method was used to activate CCC materials (PCCC, following activation). Abundant carbon–oxygen functional groups on the surface of the activated CNTs provide active sites for electrochemical reactions and significantly improve the surface capacitance of CNTs. The maximum areal capacitance was obtained when the air plasma exposure time was 20 min. The maximum areal capacitance measured as a positive electrode in a neutral solution was ∼336 mF cm^−2^ and ∼158 mF cm^−2^ as the negative electrode. The charge storage of the negative electrode is mainly based on surface-controlled capacitance, which is attributed to the adsorption capability of carbon–oxygen double bonds. When operating as the positive electrode, a large number of lithium ions intercalated in the electrode material, contributing to diffusion-controlled capacitance. The areal capacitance of the symmetrical device is 93.6 mF cm^−2^, and the areal energy density is 33 μW h cm^−2^ at a power density of 793 μW cm^−2^.

## 2. Experimental section

### 2.1. Fabrication of PCCC

Firstly, the carbon cloth (W0S1009, CeTech) was laser-cut into pieces of 1 cm × 1.5 cm and cleaned in alcohol for one day. The samples were pretreated with air plasma under an intermediate rail for 5 min to aid in the adhesion of catalysts and barrier precursor layers. Secondly, CNTs were grown on the carbon cloth *via* chemical vapor deposition (CVD), utilizing Ni(NO_3_)_2_ as a catalyst precursor and Al(NO_3_)_3_ as a barrier layer precursor. Ni(NO_3_)_2_ and Al(NO_3_)_3_ form NiO and Al_2_O_3_ at high temperatures; NiO reacts in the presence of a carbon source to form nickel particles,^41^ which act as a catalyst for carbon nanotube growth, and Al_2_O_3_ acts as a barrier layer for CNT growth.^42,43^ During the CVD process, the flow rates of C_2_H_2_, H_2_, and Ar were set to 6, 150, and 60 SCCM (standard cubic centimeters per minute), respectively. The growth time and temperature were 10 min and 700 °C. Thirdly, the obtained CNT@CC materials were treated with air plasma for 0, 10, 20, 40, and 60 min under the intermediate rail; for convenience, they were named PCCC-0 (CCC), PCCC-10, PCCC-20, PCCC-40, and PCCC-60, respectively.

### 2.2. PCCC characterization

The microstructures and morphologies of the fabricated materials were characterized using scanning electron microscopy (SEM, Tescan Mira 3). The elements present in the PCCC materials were determined *via* X-ray photoelectron spectroscopy (XPS, PHI5702). The Brunauer–Emmett–Teller (BET) surface areas of the PCCC materials were identified by nitrogen adsorption–desorption isothermals recorded at 77 K (ASAP2020, Micromeritics). Raman spectra were recorded on a Jobin Yvon HR800 micro-Raman spectrometer by a 532 nm line of a 14 mW Nd-YAG laser. The mass of each PCCC was weighed by a microbalance (Mettler, XS105DU) with a tolerance of less than 0.01 mg.

### 2.3. Electrochemical measurements and calculations

The electrochemical properties of PCCC-0, PCCC-10, PCCC-20, PCCC-40, and PCCC-60 were examined using a three-electrode cell setup (5 M LiCl, room temperature) connected to a CorrTest electrochemical station (CS310, Wuhan CorrTest Instrument Co. Ltd., China). Pt plate was used as the counter electrode, and a saturated calomel electrode (SCE) was used as the reference electrode. Cyclic voltammetry (CV) and galvanostatic charge–discharge (GCD) were performed, and the electrochemical impedance spectroscopy (EIS) results were obtained in frequencies from 100 kHz to 0.01 Hz at the open-circuit voltage with an AC voltage perturbation amplitude of 5 mV. A symmetric supercapacitor was constructed with PCCC-20 acting as both the positive electrode and the negative electrode simultaneously. The areal capacitance was calculated from the CV data using eqn (1), and from GCD plots using eqn (2):

1 *C*_s_ = *S*/(2 × *sv* × Δ*V*)

2 *C*_s_ = *I* × Δ*t*/(*s*Δ*V*)

where *C*_s_ is the areal capacitance (mF cm^−2^), *S* is the area enclosed by corresponding CV curves (A V), *s* is the area of the electrode (cm^2^), *v* is the scan rate (V s^−1^), *I* is the current, Δ*t* is the discharge time, and Δ*V* represents the potential window.

The areal energy density (*E*_s_) and areal power density (*P*_s_) were evaluated using the eqn (3) and (4):

3 *E*_s_ = *C*_s_ × Δ*V*^2^/7.2

4 *P*_s_ = 3600 × *E*_s_/*t*

where *C*_s_ is the areal capacitance (mF cm^−2^), Δ*V* is the voltage window (V), and *t* is the discharge time (s).

## 3. Results and discussion

**Fig. 1:**
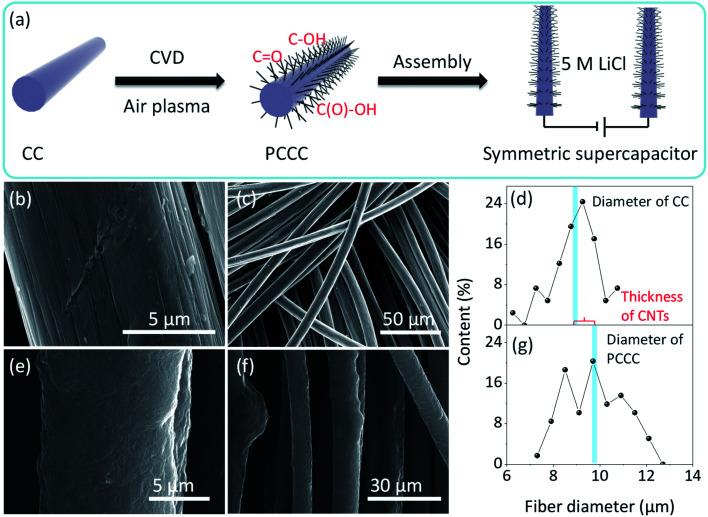
(a) Schematic diagram of PCCC fabricated and symmetric supercapacitor device (b and c) SEM images of CC at high and low magnifications (d) carbon fiber diameter in CC (e and f) SEM images of PCCC at high and low magnifications (g) carbon fiber diameter in PCCC.

Fig. 1a shows the process of CNTs growth, air plasma treatment, and assembly of the symmetrical supercapacitor. The introduction of a large number of CNTs onto the carbon fiber surface improves the specific surface area of the electrode, and then in order to enhance the pseudo-capacitance performance, an air plasma treatment was incorporated to add functional groups and defects on the surface of the carbon fiber (after ambient N_2_ and O_2_ are turned into plasma by glow discharge, the energy contained in these plasmas are greater than the energy of C=C, thus C=C are cleaved and edge-active carbon, carbon–oxygen and carbon–nitrogen functional groups are formed).^44–46^ The smooth surface morphology of pure carbon fiber in carbon cloth is shown in Fig. 1b and c; it is this smoothness that limits the specific surface area of unmodified carbon cloth electrodes, and without modification, carbon cloth does not provide many active sites for electrochemical reaction. After growing CNTs, the carbon fiber surface is uniformly covered with CNTs and remains relatively uniform on a large scale (Fig. S2†). Fig. 1d shows the diameter distribution of carbon fibers in carbon cloth before CNT growth (average diameter is ∼9 μm), and Fig. 1g shows the diameter of carbon fibers with CNTs in PCCC (average diameter is ∼9.7 μm). The average thickness of CNTs on the carbon fiber is approximately 0.7 μm, and the average diameter of CNTs is ∼19.44 nm (Fig. S1†). The presence of CNTs provides more potential sites for the formation of oxygen functional groups under plasma treatment, as well as an excellent conductive channel for electron transfer to more fully utilize the space between the carbon fibers. SEM images of PCCC are shown in Fig. 1e and f; the aggregation of carbon nanotubes occurs due to their attraction to each other after modification that includes hydrophilic functional groups, which is similar to previous reports.^38^

**Fig. 2:**
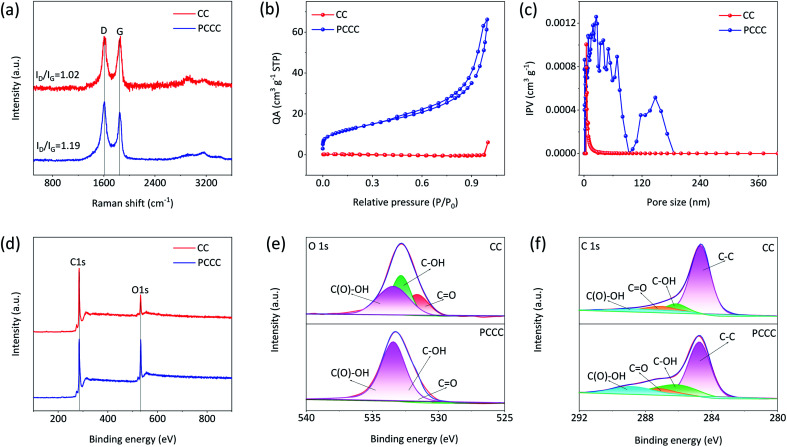
(a) Raman spectra of CC and PCCC (b) N_2_ adsorption and desorption isotherms (c) the pore size distribution of PCCC and CC (Quantity Adsorbed (QA), Inc. Pore Volume (IPV)) (d) XPS full spectra of CC and PCCC (e) O 1s and (f) C 1s XPS spectra of CC and PCCC.

In the Raman spectra of carbon materials, D-band and G-band peaks represent the defect degree and graphitization degree, respectively. Fig. 2a shows the Raman spectra of CC and PCCC; the peak intensity ratio of D-band to G-band (*I*_D_/*I*_G_) is 1.19 for PCCC, which is larger than that for CC (1.02), suggesting that the oxygen functional groups on the surface of CNTs cover or replace the graphitic structures.^47^ When employed as electrode materials in a LiCl electrolyte, the increased defect concentration can enhance the active sites for redox reactions and provide a starting point for the diffusion of Li^+^ ions. Furthermore, to identify the enhancement in the specific surface area following plasma treatment, BET analysis was performed on CC and PCCC materials. Fig. 2b and c show the N_2_ adsorption and pore size distribution of CC and PCCC, respectively. The calculated specific surface area of PCCC is 46.97 m^2^ g^−1^, which is 208 times larger than that of CC (0.24 m^2^ g^−1^), and the spatial distribution in PCCC is mainly below 100 nm, which is beneficial to the transportation of electrolyte ions. Besides, the surface of CC contains small pores (4–8 nm), which is in agreement with other studies.^48–50^ XPS was used to confirm the surface chemistry of CC, CCC, and PCCC (Fig. S3†). The wide scan XPS spectra, as shown in Fig. 2d, indicate that both oxygen and carbon elements are present in CC and PCCC. The magnified spectra of the oxygen element are shown in Fig. 2e; XPS peaks are fitted using de-convolution, assigning features to the functional groups corresponding to C=O, –COH, and –COOH groups, and the analysis mainly shows an increase in carbon–oxygen functional groups. The results indicate the successful introduction of oxygen functional groups on the surface of CNTs in PCCC. The fine spectra of carbon elements for PCCC and CC are also analyzed, as shown in Fig. 2f. There is a significant increase in C=O, –COOH, and –COH in PCCC, which is consistent with the O 1s results.^10,51^

**Fig. 3:**
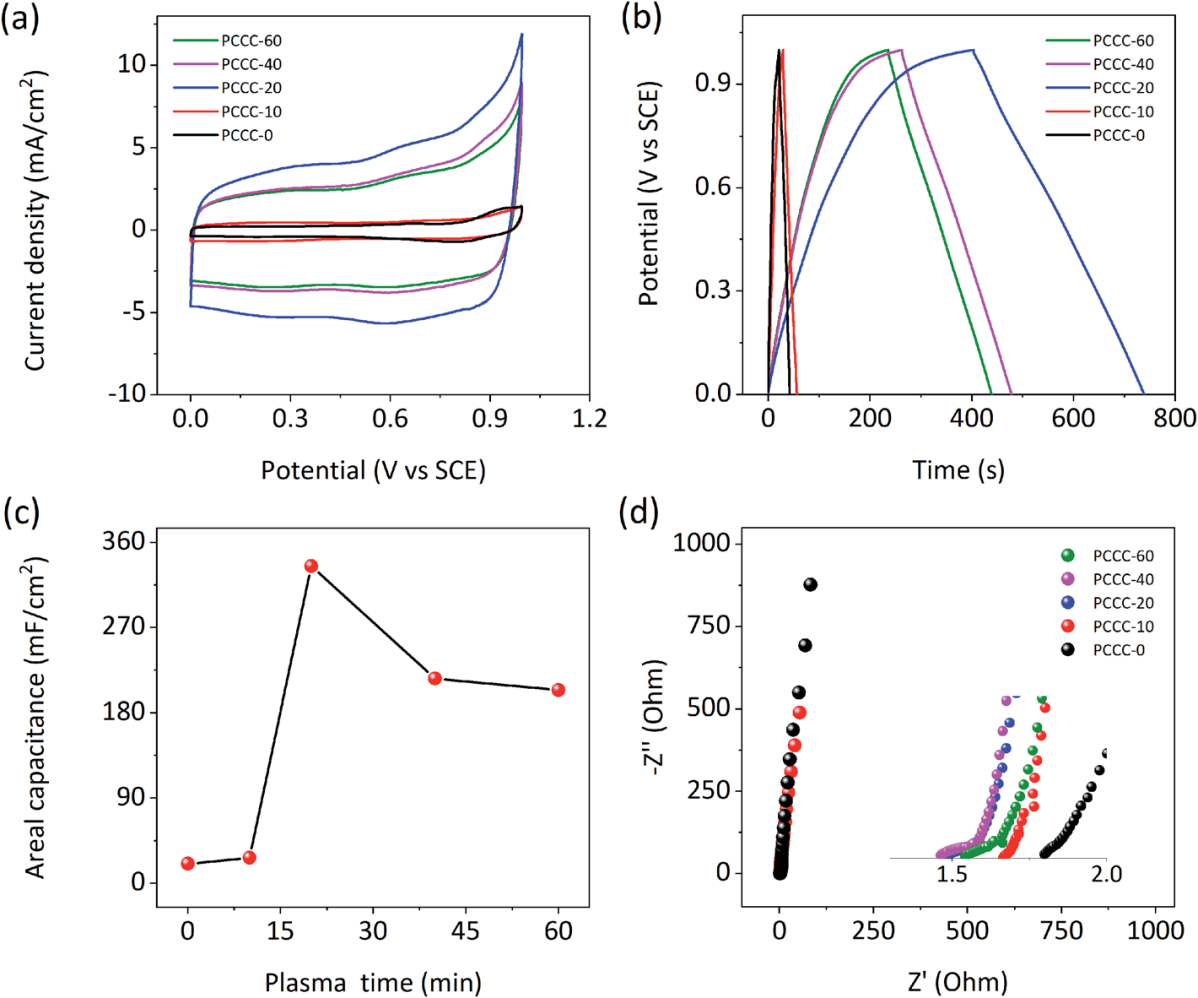
Electrochemical performance of PCCC-0, PCCC-10, PCCC-20, PCCC-40, and PCCC-60, (a) CV curves at the scan rate of 10 mV s^−1^; (b) GCD curves at the current density of 1 mA cm^−2^; (c) areal capacitance at 1 mA cm^−2^; (d) Nyquist plots.

Fig. 3a shows that the CV curves of PCCC-0, PCCC-10, PCCC-20, PCCC-40, and PCCC-60 are quasi-rectangular. When the air plasma treatment time was 20 min, the CV curve has the largest area and represents the highest areal capacitance compared with other conditions. Additionally, PCCC-20 shows a consistent rectangle at different scan rates, which indicates a better rate capability (Fig. S4†). The GCD curves of different samples are shown in Fig. 3b, and the triangular shapes clearly show their charge–discharge characteristics. GCD curves of PCCC-20 at different current densities (Fig. S5†) exhibit good rate capability. In Fig. 3c, the areal capacitance of PCCC-20 shows the best areal capacitance of 336 mF cm^−2^ at the current density of 1 mA cm^−2^, and the capacitance of PCCC-20 is ∼16 times that of PCCC-0 (∼21 mF cm^−2^). The reason that the capacitance of PCCC-10, PCCC-40, and PCCC-60 is lower than that of PCCC-20 may be that fewer oxygen functional groups do not facilitate a significant improvement in the capacitance, or too many functional groups cause the electronic transmission to be blocked and reduce the performance. In Fig. 3d, the EIS diagram shows that all the samples have smaller real axis intercept (*R*_s_) than PCCC-0, about 1.48–1.67 Ω, indicating the air plasma treatment can effectively reduce the hydrophobicity of samples and enhance the connection between the electrode and the electrolyte, which reduces the equivalent series resistance.^24,52,53^

**Fig. 4:**
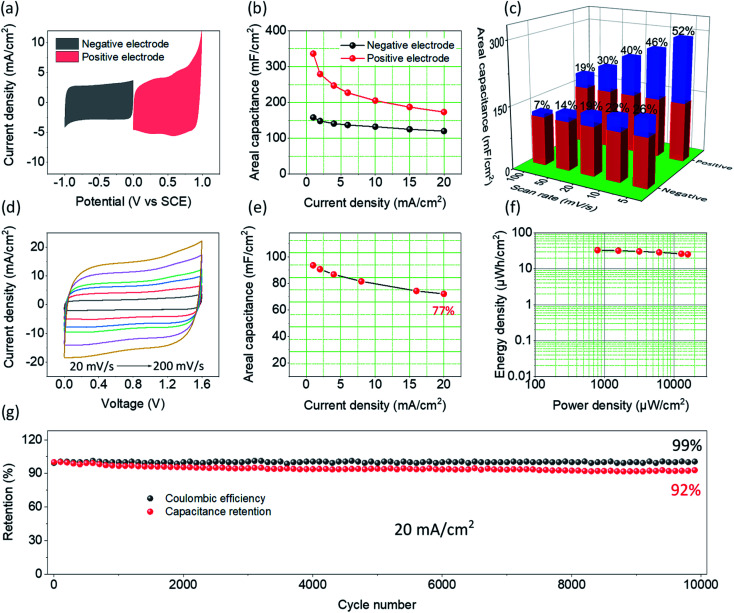
(a) CV curves of PCCC-20 as cathode and anode at scan rate of 10 mV s^−1^ (b) the areal capacitance of PCCC-20 as cathode and anode at different current densities (c) the capacitance contribution at different scan rates for PCCC as cathode and anode at different current densities (red stands for surface-controlled capacitance and blue stands for diffusion-controlled capacitance). (d) CV curves, (e) the areal capacitance, (f) Ragone plots, (g) cycling stability and coulombic efficiency of the symmetrical supercapacitor PCCC-20//PCCC-20.

To further probe the electrochemical performance of PCCC-20, a symmetrical supercapacitor was assembled. Fig. S6† shows the CV curves at different scan rates and GCD curves at different current densities, where PCCC-20 as cathode exhibits a rectangle and isosceles triangle, respectively, demonstrating capacitive behavior and excellent rate capability. Fig. 4a shows the CV curves for the carbon material as anode and cathode, both of which have a large area, which corresponds to a large capacitance. The slight polarization of the CV curve at high potentials as a positive electrode may be caused by the metal oxide doping (catalyst particles) or polar functional groups in carbon materials. In Fig. 4b, the positive and negative electrodes have a maximum capacitance of 336 mF cm^−2^ and 158 mF cm^−2^, respectively, with excellent rate capability at different current densities. In order to explain the reason why the material can be used as both positive and negative electrode simultaneously, the decomposition of surface-controlled and diffusion-controlled capacitance were elaborated using eqn (5):^54,55^

5 *i*(*v*) = *k*_1_*v* + *k*_2_*v*^1/2^

where *v* is the scan rate, *k*_1_*v* is the surface-controlled current, and *k*_2_*v*^1/2^ is the diffusion-controlled current. The surface-controlled and diffusion-controlled capacitance of PCCC-20 at different scan rates are shown in Fig. 4c. For the positive electrode, the diffusion-controlled capacitance is the main contributor to the total capacitance. The smaller lithium-ion radius can intercalate at defect sites that are produced by the plasma treatment of CNTs, and thus enhances the diffusion-controlled capacitance. As a result, the diffusion-controlled contribution reached 52% at a scan rate of 5 mV s^−1^. Because of the slow intercalation kinetics, the contribution of the diffusion-controlled capacitance decreases as the scan rate increases. Hence, the rate capability of the positive electrode is slightly lower than that of the negative electrode. For the negative electrode, the contribution of surface-controlled areal capacitance (∼74% at a scan rate of 5 mV s^−1^) is mainly due to the reaction center contributed by the plasma treatment, which enhances the capacitance through eqn (6) and (7):^56–58^

6 –C–OH ↔ –C=O + H^+^ + e^−^

7 –C(O)OH ↔ –COO + H^+^ + e^−^

To better understand the electrochemical behavior of PCCC-20 as a positive and negative electrode in a real supercapacitor, a symmetric device (PCCC-20//PCCC-20) was constructed. The supercapacitor-dependent CV curves are shown in Fig. S7† at a scan rate of 20 mV s^−1^, and the results indicate that the stable operational voltage is 1.6 V without apparent polarization. As shown in Fig. 4d, CV curves of the supercapacitor exhibit a regular rectangular shape, and even at a scan rate of 200 mV s^−1^, the curves still have an excellent rectangular shape, indicating capacitive behavior and excellent rate capability. Fig. 4e also shows that the supercapacitor has good rate capability at different current densities (Fig. S8†). When the current density increases from 1 to 20 mA cm^−2^, the areal capacitance of PCCC-20//PCCC-20 retains 77% of its original value (from 93.6 mF cm^−2^ to 72.1 mF cm^−2^), indicating excellent electrochemical performance as a supercapacitor. Furthermore, the areal capacitance of the supercapacitor outperforms most reported results, such as dc-pulse nitrogen atmospheric-pressure plasma jet calcined CNT-coated carbon cloth (6.10 mF cm^−2^),^38^ CNT-PDMS sponge (13.8 mF cm^−2^),^59^ PPy@CNTs@urethane elastic fiber (69 mF cm^−2^),^60^ CNT-incorporated tin-oxide (21 mF cm^−2^),^61^ and rGO/Ni pattern (12.5 mF cm^−2^).^62^ The symmetric supercapacitor displays a maximum energy density of 33 μW h cm^−2^ at 793 μW cm^−2^, as shown in the Ragone plot in Fig. 4f. When the power density increases to 15 911 μW cm^−2^, the energy density remains as high as 26 μW h cm^−2^, indicating good energy storage performance. A test of the cyclic stability of supercapacitors is shown in Fig. 4g; after a 10 000 cycle test, the areal capacitance remains at ∼92%, and the coulombic efficiency is ∼99%, demonstrating the excellent potential for supercapacitance.

## 4. Conclusion

In summary, we use an efficient air plasma method to modify CNTs with oxygen functional groups. This strategy enhanced the electrochemical active sites and defects on the surface of CNTs and increased their areal capacitance and their potential as a bipolar electrode. Compared with untreated CNTs (PCCC-0), the capacitance of PCCC-20 increases by 16 times. At a current density of 1 mA cm^−2^, the areal capacitance of the positive electrode is 336 mF cm^−2^, and 158 mF cm^−2^ when used as a negative electrode. The main reasons for the excellent performance are as follows: (a) the oxygen functional groups produced by air plasma treatment increases the number of active sites; (b) air plasma treatment defects provide an entry point for the shallow intercalation of lithium ions. Furthermore, the PCCC electrode used for the preparation of the PCCC-20//PCCC-20 symmetric supercapacitor shows a good areal capacitance of 93.6 mF cm^−2^, high rate capability, a high energy density of 33 μW h cm^−2^, and a high power density of 15 911 μW cm^−2^. Finally, the device also exhibits excellent cyclic stability, as evidenced by the loss of only ∼8% of starting capacitance after 10 000 cycles. This work helps in understanding the energy storage process of carbon materials at both positive and negative electrodes, and further provides ideas for large-scale manufacturing of all carbon-based supercapacitors.

## Conflicts of interest

There are no conflicts to declare.

## Supplementary Material

RA-010-D0RA06888H-s001
