# Supplementary material for: Boosting the electrochemical properties of carbon materials as bipolar electrodes by introducing oxygen functional groups
Source: RSC Adv. 2020 Sep 24;10(58):35295–301. doi: 10.1039/d0ra06888h (PMC9056941; doi:10.1039/d0ra06888h)
Supplement: RA-010-D0RA06888H-s001 [file RA-010-D0RA06888H-s001.pdf]

Electronic Supplementary Material (ESI) for RSC Advances.
This journal is © The Royal Society of Chemistry 2020

Supporting information

Boosting the Electrochemical Properties of Carbon Materials as Bipolar Electrodes by Reasonable Oxygen Functional Groups

Yaxiong Zhang[a], Ying Liu[b], Yunfei Bai[c], Yupeng Liu[a], Erqing Xie[a]*

[a] Key Laboratory for Magnetism and Magnetic Materials of the Ministry of Education, School of Physical Science and Technology, Lanzhou University, Lanzhou 730000, China

[b] School of Physics and Optoelectronic Engineering, Ludong University, Yantai 264025, China

[c] State Key Laboratory of Applied Organic Chemistry, Key Laboratory of Nonferrous Metal Chemistry and Resources Utilization of Gansu Province, College of Chemistry and Chemical Engineering, Lanzhou University, Lanzhou 730000, China

Corresponding authors. E-mail: xieeq@lzu.edu.cn (E. Xie)

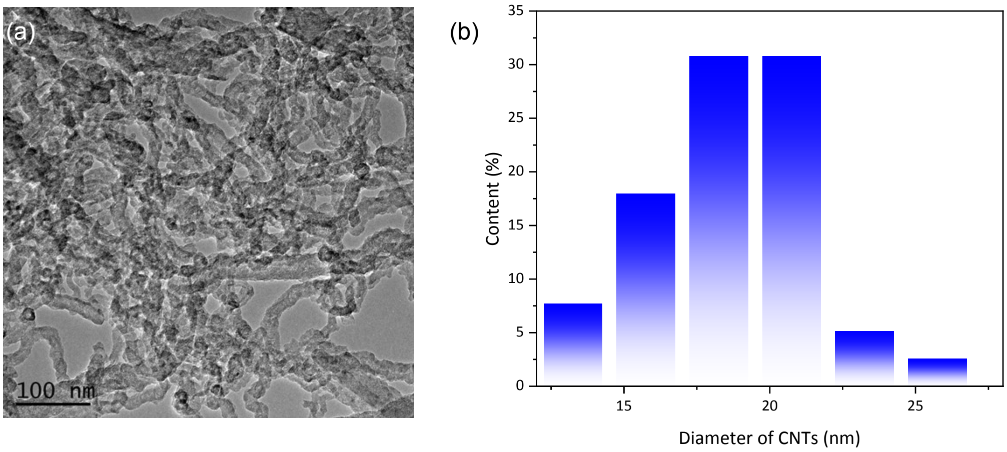

Figure S1 TEM image and average diameter of CNTs.

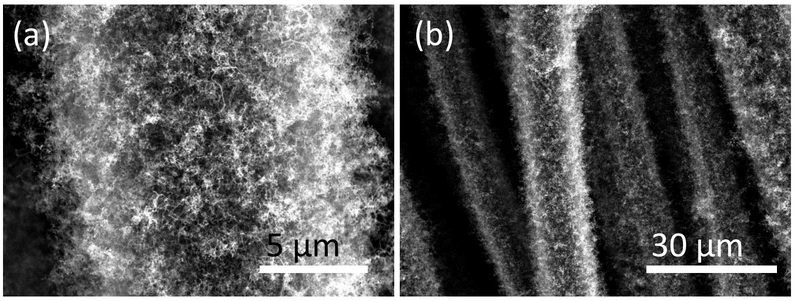

Figure S2 The SEM images of CCC

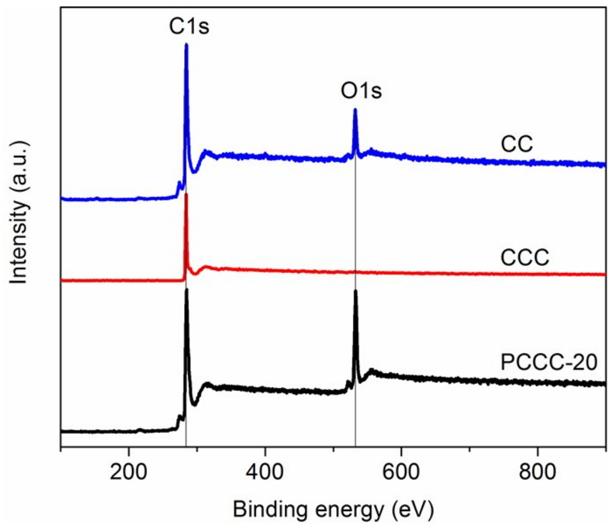

Figure S3 XPS wide scan spectra of CC, CCC, and PCCC

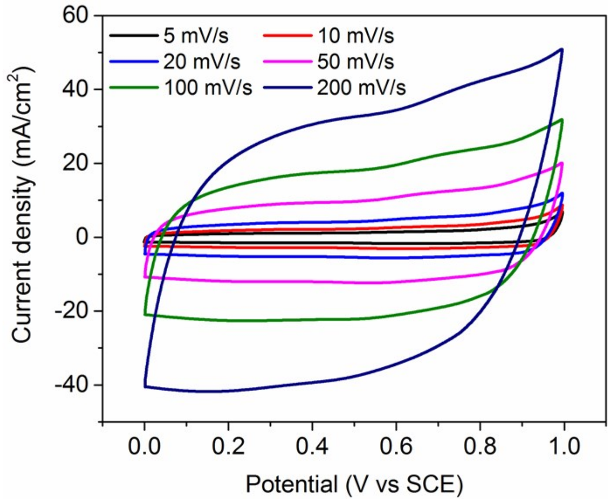

Figure S4 CV curves of PCCC-20 at different scan rates

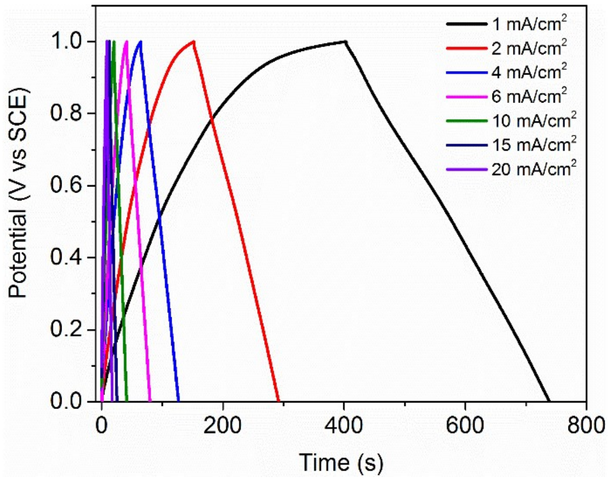

Figure S5 PCCC-20 as anode, the GCD curves at different current densities.

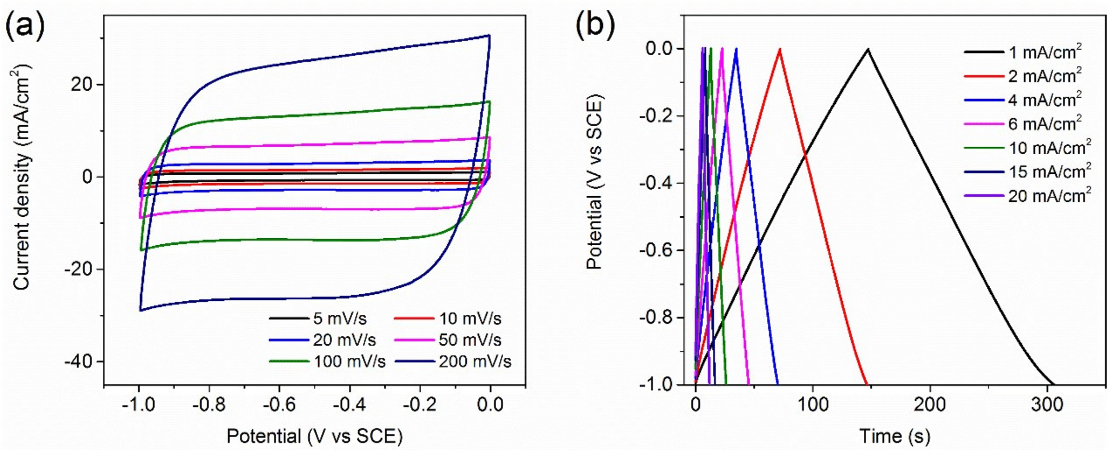

Figure S6 PCCC-20 as cathode (a) the CV curves at different scan rates and (b) the GCD curves at different current densities.

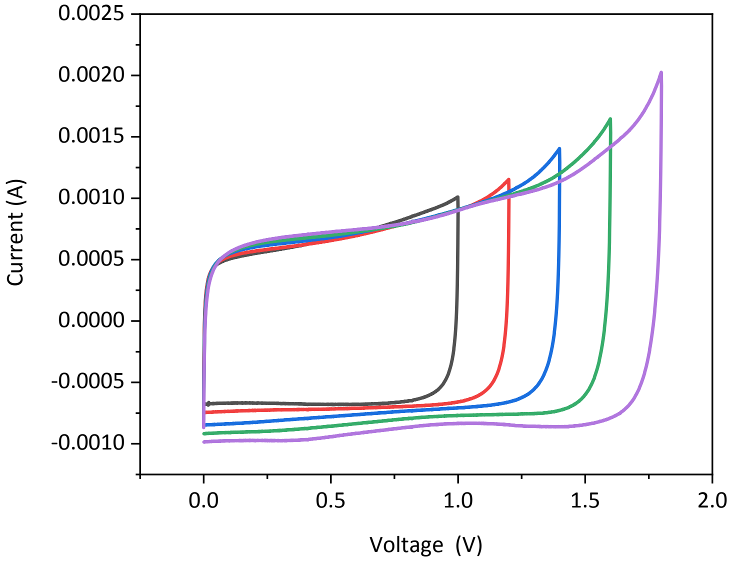

Figure S7 CV curves of the supercapacitor at different voltage windows at a scan rate of 20 mV/s.

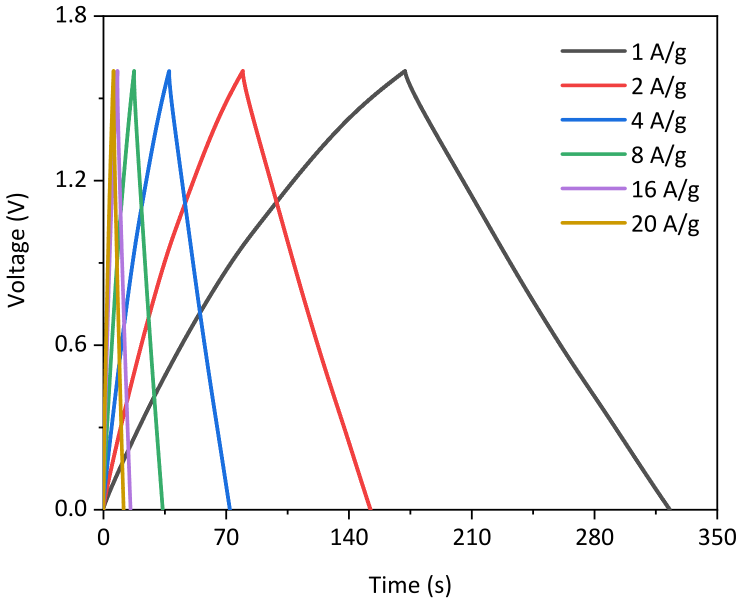

Figure S8 GCD curves of the supercapacitor at different current densities.
